# An analysis of interprovincial migration in Vietnam from 1989 to 2009

**DOI:** 10.3402/gha.v5i0.9334

**Published:** 2012-12-31

**Authors:** Le Thi Kim Anh, Lan Hoang Vu, Bassirou Bonfoh, Esther Schelling

**Affiliations:** 1 Department of Epidemiology and Biostatistics, The Hanoi School of Public Health, Hanoi, Vietnam; 2 Swiss Tropical and Public Health Institute and University of Basel, Basel, Switzerland; 3 Centre Suisse de Recherches Scientifiques en Côte d'Ivoire, Abidjan, Ivory Coast

**Keywords:** Vietnam, interprovincial migration, trends, urbanization, migration rates, census

## Abstract

**Background:**

In Vietnam, reports either present general patterns of internal migration or the migration characteristics of specific subgroups. Reports are often based on small numbers and do not examine the relationships between socioeconomic factors and migration. Different reports classify migrant populations differently, presenting difficulties for researchers and policymakers to gain a consistent picture of migration (particularly of interprovincial migration) and limiting the ability of policymakers to plan services appropriately. This study describes the characteristics of all migrants in Vietnam, focusing on interprovincial migrants, and examines age and sex trends and correlations among in-migration, urbanization, and individual income.

**Methods:**

We analyzed data from the 15% sample survey in the 2009 Population and Housing Census, the 3% sample in the 1999 national census, the 5% sample in the 1989 national census, and selected data from the 2008 Vietnam Household Living Standards Survey. Logistic regression was used to identify socioeconomic factors related to migration.

**Results:**

In 2009, of 6.7 million internal migrants (approximately 6.5% of the total population), 3.4 million were interprovincial migrants. Three notable trends were observed between 1989 and 2009: (i) the total population is characterized by increasing proportions of migrants; (ii) the proportion of female migrants is growing; and (iii) the average age of migrants is decreasing. Socioeconomic factors related to interprovincial migration include provincial economic status (monthly income per capita: OR = 4.62, p = 0.005) and urbanization (proportion of urban population: OR = 3.47, p = 0.019), suggesting that provinces with high monthly income per capita and urbanization are more likely to have higher rates of in-migration.

**Conclusion:**

These findings reflect the effects of unequally growing labor markets in Vietnamese provinces on migration, and are suggestive of infrastructure improvements and public service needs in these areas. Analysis of migration can provide useful information for planning health and social services and for policymaking for national economic development.

Internal migration is inevitable in countries that undergo rapid economic and social development (1). China, Indonesia, India, Thailand, and other Asian nations have recently experienced dramatic increases in internal migration, particularly in interprovincial migration (2, 3). This kind of migration has greatly reduced poverty by meeting labor demands but bears risks for migrants and their home communities due to limited access to social services and information about employment, welfare, and health (2, 3).

In Vietnam, the transformation from a centrally planned economy with public ownership of production to a market economy that encouraged individual entrepreneurship and foreign investment (renovation, or *Doi Moi* in Vietnamese) began in 1986 and has led to significant economic growth and poverty reduction. However, the benefits of *Doi Moi* have been unequally distributed among regions: while cities such as Ho Chi Minh City and Hanoi as well as surrounding provinces have received large levels of industrial capital, the northern mountains, north central coast, central highlands, and other rural areas have lagged behind. These disparities have triggered a flow of rural-to-urban migration (1, 4).

**Policy Recommendations**

To have better supports to the lives of internal migrants – especially rural-to-urban migrants – in Viet Nam it is necessary to understand trends and characteristics of the migrants and factors promoting rural-to-urban migration. The following recommendations are key points for that drawn from this study:

- National policy needs to identify rural-to-urban migrants as an important human resource for development of national industrial zones.
- Cities/Provinces with large industrial zones or national projects need to improve their basic infrastructure such as accommodation with good living conditions, local health system, and schools for migrants’ children before opening for migrants.
- Cities/Provinces with large industrial zones or national projects need to have specific policies for supporting migrants. These policies need to aim ensuring the rights of migrants in accessing social welfare and health insurance.
- National programs (e.g. health programs) related to migrants need to focus on young migrants, especially female migrants. These programs need to recognize female migrants as priority targets due to predominant proportion of this group.

Researchers have noted that migration enables access to employment, providing better wages for people from rural areas (5, 6). Migration is not only a way for migrants to better their lives but is also a significant source of family support via remittances (2, 3, 5, 7). For most migrants, movement is not due to unemployment but is prompted by a desire to improve working, income, and living conditions for themselves and their families. The 2004 Vietnam Migration Survey stated that economic motives account for the moves of more than 70% of all types of migrants (8).

Difficulties of definition characterize research on migration. Related concepts (e.g. temporary, seasonal, permanent, or floating migration) are used interchangeably and the indicators presented have been controversial (9–11). Indeed no uniform definition of migration is apparent in the literature. This is because migration involves a temporal and spatial context. In addition, migration studies typically employ different definitions in conjunction with different types of data (6). According to the Joint United Nations Programme on HIV/AIDS, migrants are ‘people who move from one place to another temporarily, seasonally, or permanently for a host of voluntary and/or involuntary reasons’ (12). This definition incorporates internal or domestic migration, which refers to the movement of people from one place to another within the same country. Due to difficulties in defining types of population mobility, it is important to have a clear terminology for the Vietnamese context when looking into problems that include access to health care for mobile populations within that country.

In Vietnamese migration-related policy documents, the government classifies migrants according to two groups: organized/sponsored/government-controlled migrants (i.e. people who migrate within the country and are directed by government plans due to the loss of land or natural calamities, or who volunteer to go to a new economic zone) and spontaneous migrants (i.e. people who migrate within the country but who are not organized by the government) (13). Relative to organized migrants, spontaneous or voluntary migrants are likely to be more vulnerable in terms of social protection because they are recognized but neither encouraged or supported, nor are they authorized. Despite an enhanced vulnerability, nowadays most internal migrants are spontaneous (1).

In the 2009 Population and Housing Census, internal or domestic migrants are identified according to one of three categories: interprovincial migrants—persons moving from one province to another within the country; interdistrict/intraprovincial migrants—persons moving from one district to another within a province; and intradistrict migrants—persons moving within a district (14, 15). People who move spontaneously for work generally prefer areas nearer their homes in order to maintain links and benefit from the mutual support of their families. By contrast, interprovincial migrants—especially migrants moving without their families—do not have such access to family support and are likely to be the most vulnerable group among internal migrants (10).

In Vietnam, reports tend to present either general patterns of internal migration or characteristics of specific subgroups, such as long-distance truck drivers, sexual workers, construction workers, seafarers, and laborers (16–18). These reports are commonly based on small numbers and do not examine the relationships between socioeconomic factors and migration. In addition, reports use different classifications of migration, making it difficult for researchers and policymakers to gain a consistent picture, particularly regarding interprovincial migration.

This study attempts to address these gaps by drawing on successive national census data sets with the following research aims: (i) to describe the main characteristics of the interprovincial migration population in Vietnam; (ii) to capture the trends of interprovincial migration over time; and (iii) to examine correlations between provincial socioeconomic status (SES) and internal migration rates. This project is part of a larger study on ‘access to health care services, health risks, and resilience of interprovincial migrants in Vietnam’ (19, 20).

## Methods

### Data sources

The analysis used raw data from the 15% sample survey in the 2009 Population and Housing Census, the 3% sample survey in the 1999 national census, and the 5% sample survey in the 1989 national census of Vietnam (21–23). These survey samples were selected using multistage cluster sampling. Technical issues of the sampling procedure were clarified by the Vietnam General Statistical Office (GSO) in *The 2009 Vietnam Population and Housing Census: Implementation and Preliminary Results* (24). Provincial SES indicators were calculated based on data from the 2008 Vietnam Household Living Standards Survey (25), and the provincial urbanization rate was calculated from the 2009 national census data.

### Definitions of migration indicators

In each national census, a person was considered a migrant if the current place of residence at the time of survey and the place of residence 5 years prior were not the same commune (i.e. smallest administrative unit). The term ‘interprovincial migrant’ used in this study therefore refers to the population who changed provinces during the previous 5 years of the census. Based on the rural and urban characteristics of place of residence 5 years prior to the census versus those of the current place of residence, migration flows were identified according to four categories: rural-to-rural, rural-to-urban, urban-to-urban, and urban-to-rural migration.

Classification of an area as urban or rural was based on the National Administrative Map (26, 27). Urbanization is understood quantitatively—reflecting a growing population, increased population density, territorial expansion, and production development and qualitatively—indicating changes in living standards and diversification of socioeconomic cultural patterns (28). However, among Third World nations, urbanization is largely taking place in the sense of expanding breadth of urban areas and in terms of population growth. In this study, we therefore consider the proportion of the population living in urban areas (i.e. urban population) to indicate the degree of urbanization: the ‘urban population is composed of residents of cities, provincial towns, and district townships’ (29).

The in-migration rate refers to the number of people arriving from other provinces during an observed period per 1,000 persons aged 5 and above in the destination province. The out-migration rate refers to the number of migrants departing from one province during an observed period per 1,000 persons aged 5 and above in that province.

The net gain of the population through migration refers to the mean of the absolute in-migration population per year during the period 2004–2009 in a given area. Subsequently, the net loss of the population through migration is the mean of the absolute out-migration population per year during the same period in a given area.

In addition, in the 2009 census, the main construction materials of the pier, roof, and outer walls were used to categorize housing status according to one of three levels: permanent, semi-permanent, and simple. Hygienic toilet facilities were defined as ‘flush toilets with septic tanks and sewage pipes’ and safe water was defined as ‘coming from an indoor or public tap, drilled well water, a protected dug well, or rainwater.’ These indicators were utilized from *The 2009 Vietnam Population and Housing Census: Some Key Indicators* (30).

### Statistical analysis

Data used for analysis were extrapolated from the census sample using expansion factors (weights) provided by the Vietnam GSO because the sample captured only 15% of total population. As stated earlier, all information regarding technical issues related to the census data is contained in *The 2009 Vietnam Population and Housing Census: Implementation and Preliminary Results* (24).

In this analysis, tabular techniques were used to present the characteristics of the internal migrant population. The maps in the report relied on data disaggregated by province to show migration flows and distribution of industrial zones in Vietnam. In addition, crude odds ratios calculated by a simple logistic regression were used to identify factors related to in-migration. These correlates included provincial economic status (e.g. monthly income per capita, poverty rate, and the ratio of fifth to first wealth quintile), social status (e.g. school dropout and unemployment rates), and urbanization (e.g. proportion of population classified as urban). Due to an empirical correlation between the poverty rate and monthly income per capita, monthly income per capita and the ratio of fifth to first quintile were adopted as indicators of economic status. Moreover, because all the selected variables had non-normal distributions and were continuous variables, their medians were used to convert them into categorical variables. According to this approach, continuous variables were classified as ‘low’—below median and ‘high’—above median (e.g. in-migration rate was divided into two categories: low and high in-migration rate).

## Results

Of the 6.7 million internal migrants in 2009 (including intradistrict, interdistrict, and interprovincial migrants), accounting for 6.5% of the total population, approximately 3.4 million were interprovincial migrants. Interprovincial migrants were mostly young, with a median age of 24 and 23 for males and females, respectively. Compared with interdistrict and intradistrict migrants, the interprovincial migrants significantly were the youngest (Table 1).

**Table 1:** Main characteristics of *internal migrants* aged 5 years and older in Vietnam, National Census, 2009

Characteristics	Interprovincial migrants	Interdistrict migrants	Intradistrict migrants
Number of internal migrants	6,724,960		
Number of migrants (%)	3,397,904 (50.5%)	1,708,896 (25.4%)	1,618,160 (24.1%)
Age (median)	24	25	26
Male	24	27	29
Female	23	24	25
Gender (%)			
Male	47.0	43.4	36.4
Female	53.0	56.6	63.6
Housing status (%)			
Simple	28.5	27.1	28.1
Semi-permanent	48.5	41.2	40.5
Permanent	23.0	31.6	31.4
Safe water for drinking and cooking (%)	94.5	92.0	86.8
Toilet facilities (%)			
None	3.4	4.1	7.0
Other (non-hygienic toilet)	13.4	17.4	30.5
Hygienic toilet	83.3	78.5	62.6

All differences among migrant populations are statistically significant with p < 0.001.

Table 1 also shows that only 23% of the interprovincial migrants lived in permanent houses with concrete roofs, while 48.5% lived in semi-permanent houses with tile or tin roofs, and 28.5% lived in simple houses with leaf, or straw-oil paper, roofs. The proportion of interprovincial migrants living in permanent housing was the lowest of all other migrant groups. However, a high proportion used safe water, and hygienic toilets were observed for all types of migrants.

The proportion of females is higher than that of males among migrant populations (Table 2). The ratio of female/male migrants is highest for rural-to-rural migration, followed by rural-to-urban, urban-to-urban, and urban-to-rural migration. Moreover, analysis of the 2009 census data reveals that females accounted for more than half of the interprovincial migrant population in all but one type of migration (the exception is urban-to-rural migration in the interprovincial group, but the female proportion was very close to 50%) (Table 3). In addition, Tables 2 and 3 show that 20- to 40-year-olds made up the highest proportion of the total migrant population and that the concentration of young adults among migrants was also highest for rural-to-urban migration.

**Table 2:** Main characteristics of *internal migrants* aged 5 years and older, comprising 8.6% of the whole population, by migration flows and using Vietnam National Census data, 2009

Characteristics	Rural-to-rural	Rural-to-urban	Urban-to-urban	Urban-to-rural
Proportion of migrants (%)	33.7	31.6	26.3	8.4
Age (median)	24	23	28	27
Age groups (%)				
Under 20 years	22.3	24.8	20.1	17.3
20–40 years	67.2	66.7	59.9	63.6
Over 40 years	10.5	8.5	20.0	19.1
Gender (%)				
Male	39.3	44.8	46.3	48.4
Female	60.7	55.2	53.7	51.6
Ethnic group				
Kinh	84.3	95.2	96.0	93.9
Tay	2.4	1.1	0.6	1.4
Thai	2.7	0.7	0.1	0.4
Muong	2.0	0.6	0.1	0.6
Khomer	1.5	0.9	0.2	0.5
Hmong	1.4	0.1	0.1	0.3
Others	5.7	1.4	2.9	2.9
Education				
Illiterate	4.2	1.1	1.2	2.2
Less than primary school	14.2	6.9	10.7	13.6
Primary school	25.9	16.6	15.5	21.2
Secondary school	25.5	21.5	15.8	20.5
High school	18.2	35.9	22.7	15.7
Primary vocational training	2.4	3.7	3.7	3.8
Intermediate vocational training	4.9	6.1	7.4	8.9
Vocational junior college	0.3	0.4	0.5	0.7
Junior college	1.9	1.9	2.7	3.3
Graduate	2.5	5.8	18.5	9.8
Postgraduate	0.0	0.1	1.3	0.3

Note: All differences among migrant populations are statistically significant with p < 0.001.

**Table 3:** Main characteristics of interprovincial migrants aged 5 years and older, comprising 4.3% of the whole population (and 50.5% of the internal migration population), by migration flows and using Vietnam National Census data, 2009

Characteristics	Rural-to-rural	Rural-to-urban	Urban-to-urban	Urban-to-rural
Proportion of migrants (%)	35.4	44.5	7.3	12.8
Age (median)	24	23	24	26
Age groups (%)				
Under 20 years	24.0	24.7	21.0	16.2
20–40 years	66.1	67.1	67.5	67.3
Over 40 years	9.9	8.1	11.5	16.5
Gender (%)				
Male	46.1	46.6	48.1	51.5
Female	53.9	53.4	51.9	48.5
Ethnic group				
Kinh	89.0	96.4	96.9	94.8
Tay	1.8	0.7	1.0	1.4
Thai	0.9	0.3	0.2	0.3
Muong	2.0	0.6	0.3	0.8
Khomer	1.7	1.0	0.3	0.6
Hmong	1.1	0.0	0.0	0.1
Others	3.4	1.1	1.3	1.9
Education				
Illiterate	3.2	0.9	0.7	1.8
Less than primary school	12.4	6.4	6.2	11.8
Primary school	26.5	17.8	11.8	20.2
Secondary school	26.7	23.5	14.1	22.5
High school	21.6	35.8	36.4	18.5
Primary vocational training	2.5	3.8	3.2	3.7
Intermediate vocational training	4.0	4.9	7.2	8.9
Vocational junior college	0.3	0.4	0.5	0.8
Junior college	1.1	1.5	2.6	2.6
Graduate	1.7	4.9	16.6	9.0
Postgraduate	0.0	0.1	0.7	0.2

Note: All differences among migrant populations are statistically significant with p < 0.001.

A high proportion of migrants with high school education or higher was observed for interprovincial groups. This is similar to the trend for the total internal migrant population. The proportion was highest for urban-to-urban migrants (57.2%), followed by rural-to-urban migrants (51.4%), rural-to-rural migrants (31.2%), and urban-to-rural migrants (31.0%) (Table 2). The Kinh people accounted for more than 90% of all migrants, while other ethnic minorities made up low proportions of internal and interprovincial migrants, mostly engaging in rural-to-rural migration (Tables 2 and 3).

**Fig. 1:**
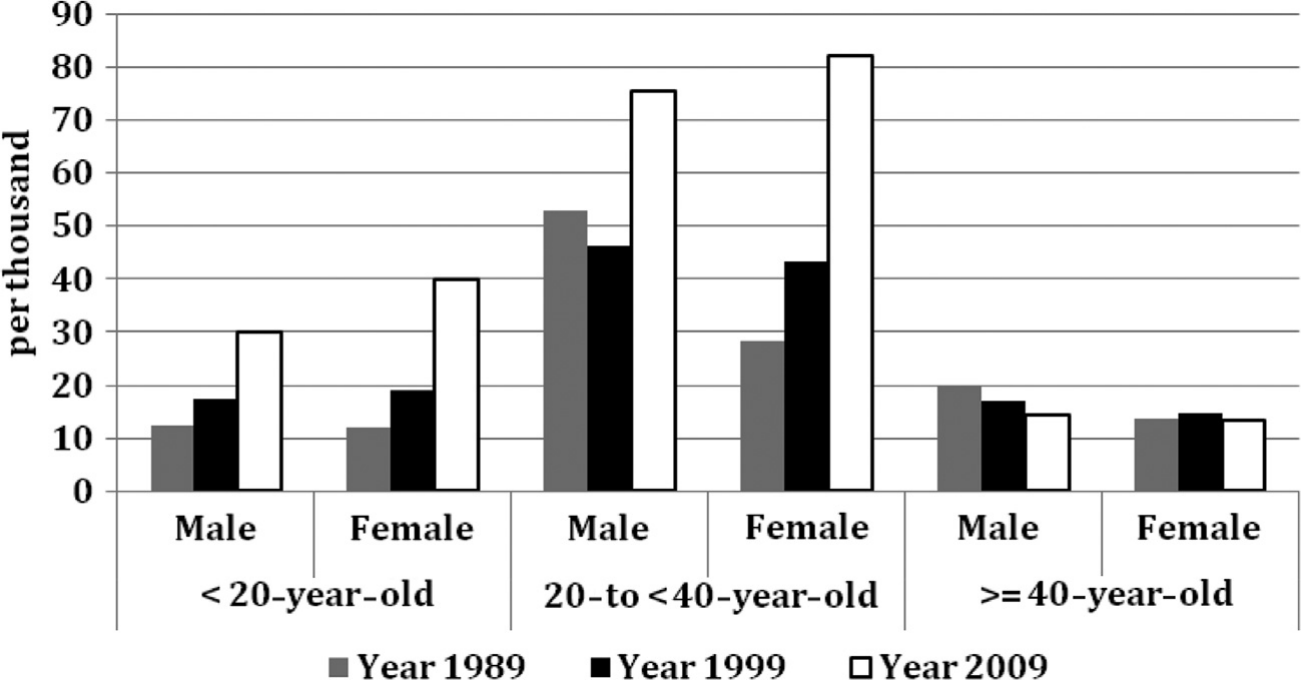
Trends of interprovincial migration rates (migrants per 1,000 people) over time by gender and age groups. All proportions presented in this graph were estimated using census data with an extremely large sample size; the standard deviation of the proportion is extremely small (such as 0.007%) and the 95% CI extremely narrow, and thus the 95% CI was hardly presented in the chart.

As shown in Fig. 1, interprovincial migration has increased over time. The population of migrants (as defined in this study) increased from 1.3 million in 1989 (2.5% of the total population) to 2 million in 1999 (2.9% of the total population) and to 3.4 million people in 2009 (4.3% of the total population). The relative figures indicate a proportional increase of internal migration, particularly between 1999 and 2009.

Increasing female migration rates are also observed. In 1989, the migration rate of females was lower than that of males for all age groups. However, in 2009, the migration rates among females were higher compared with the rates of males for all age groups, and this difference was most pronounced among the youngest age group (i.e. for those aged 20 and under, the migration rate was 40/1,000 for women versus 30/1,000 for men).

The interprovincial migrant population has also become, on average, younger over time. The median age of internal male and female migrants in the 1999 census was 25 years for both sexes. However, in the 2009 census, the median age dropped to 24 years for men and 23 years for women. Fig. 1 also shows that the migration rates of men and women under the age of 20 doubled and trebled, respectively, between 1989 and 2009.

Rates of migration also vary across provinces. In some provinces, the migrant population represents more than 10% of the total population, such as in Ho Chi Minh City (HCMC), Binh Duong, and Hanoi. By contrast, the migrant population in other provinces accounts for less than 1% of the total population.

**Fig. 2:**
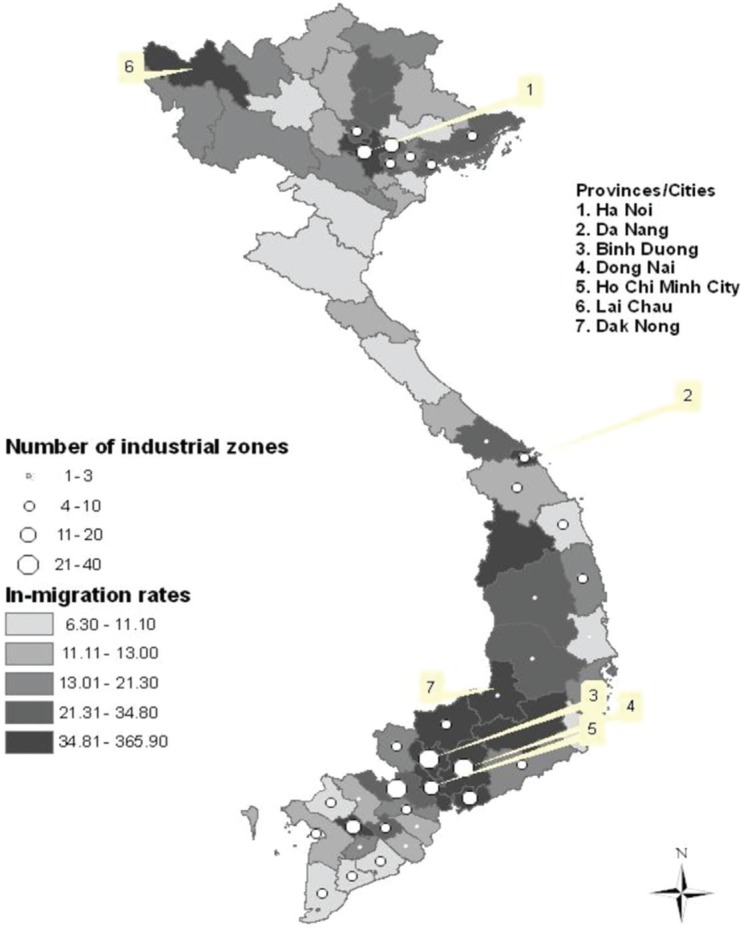
In-migration rates and number of industrial zones by province in Vietnam, 2009, excluding Hoang Sa and Truong Sa islands. The map shows the number of industrial zones but does not show the extent of the industrial zones. Note: Distribution of variables in this figure is skewed; thus, these variables are classified into categories by using the quintile.

**Fig. 3:**
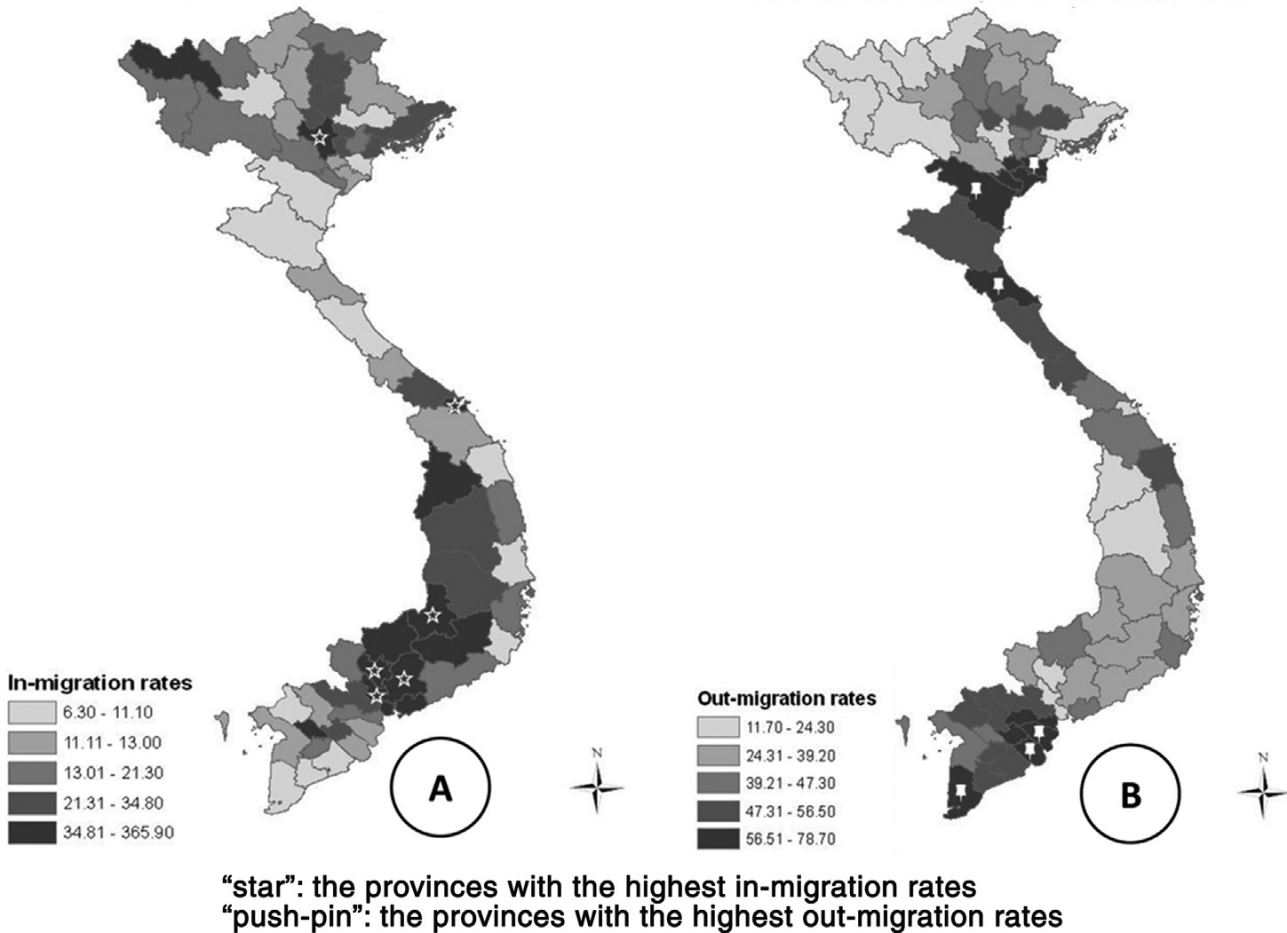
Migration rates in Vietnam, 2009, excluding Hoang Sa and Truong Sa islands. Part A shows the in-migration rates by province, and part B shows the out-migration rates by province. Note: Distribution of variables in this figure is skewed; thus, these variables are classified into categories by using the quintile.

Figs. 2 and 3A illustrate the geographical migration flows. The provinces with high in-migration rates are mostly concentrated in the southeast—a region that is considered to be the economic center of Vietnam. The first five provinces/cities (numbered 1–5 in Fig. 2) are provinces/cities that have a large number of industrial zones and high in-migration rates. Their large industrial cities (e.g. Hanoi, Da Nang, Binh Duong, Dong Nai, and Ho Chi Minh City) have attracted huge annual migration flows from other regions. For instance, the net gain of the population through migration has been nearly one million people in Ho Chi Minh City and half a million people in Binh Duong. However, Lai Chau (in the northwest) and Dak Nong province (in the central highlands) have also experienced very high in-migration rates though these provinces have no or only one industrial zone (Fig. 2).

Meanwhile, the Red River Delta area (e.g. Thai Binh province) and the north central area (e.g. Thanh Hoa and Ha Tinh provinces) have experienced a net loss of almost 100,000–200,000 people per province. Together with the Mekong River Delta area (e.g. Ben Tre, Tra Vinh, and Ca Mau provinces), these provinces had the highest out-migration rates (Fig. 3B).

**Fig. 4:**
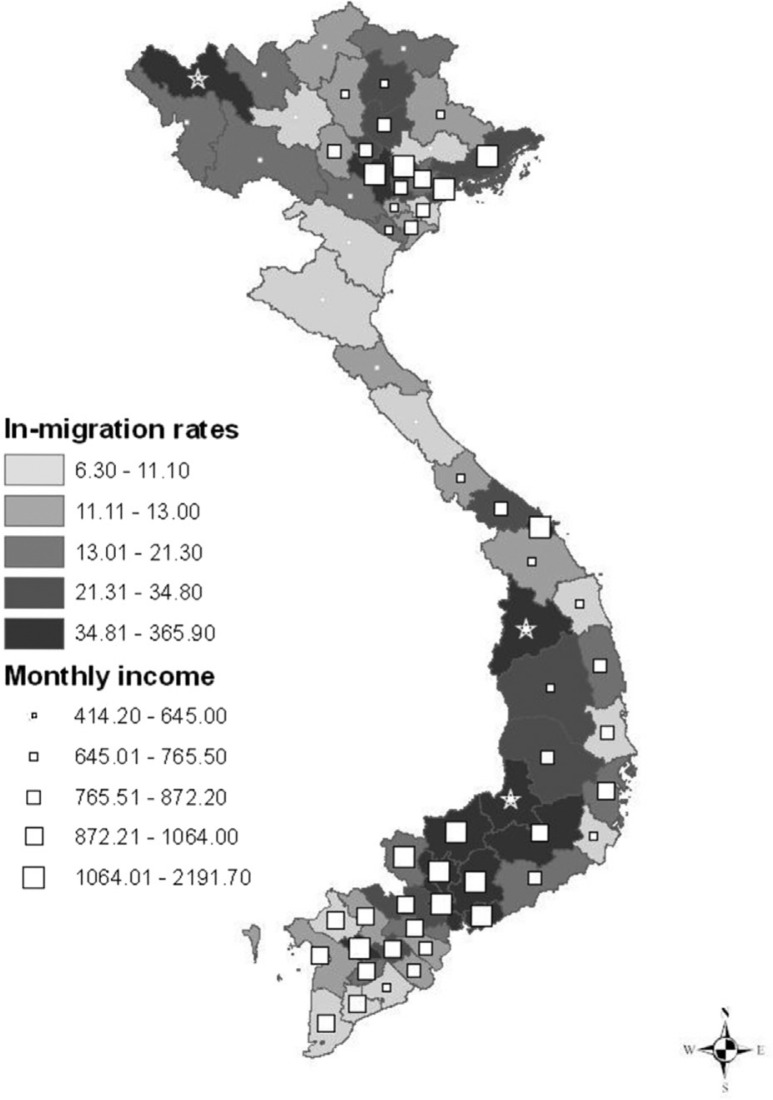
The relation between migration and monthly income per capita in the Vietnamese population, excluding Hoang Sa and Truong Sa islands. The ‘star’ symbols in the top northeast corner and the two ‘star’ symbols in the east middle show the Lai Chau, Kon Tum, and Dak Nong provinces, which are outliers with high in-migration rates and low monthly per capita income. Note: Distribution of variables in this figure is skewed; thus, these variables are classified into categories by using the quintile.

Moreover, a simple logistic regression was used to capture correlates of in-migration. Table 4 presents the crude odds ratios of in-migration and its correlates, such as monthly income per capita and proportion of urban population. This analysis illustrates that provinces with high monthly income per capita (i.e. above 803,000 Vietnam dong [VND]/month) and a high proportion of urban population (i.e. above 19.1% of the whole population) are more likely to have higher in-migration rates than other provinces (OR = 4.62 and OR = 3.47, respectively). By contrast, factors such as unemployment rate, school dropout rate, and the ratio of fifth to first quintile appear not to be related to in-migration (Fig. 4).

**Table 4:** Crude odds ratios of in-migration and its correlates

	OR	SE	95%CI of OR		p
Monthly income per capita (>803,000 VND/month)	4.62	2.5	1.6	13.35	0.005
Unemployment rate (>2.1%)	0.94	0.47	0.35	2.52	0.898
Proportion of urban population (>19.1%)	3.47	1.83	1.23	9.78	0.019
School dropout rate (>15.4%)	0.56	0.29	0.21	1.52	0.258
Ratio of fifth to first quintile (>6.68%)	1.06	0.54	0.4	2.86	0.904

## Discussion

The 2009 national census used administrative boundaries (i.e. commune/ward, district, and province) to divide internal migration into three categories: intradistrict, interdistrict, and interprovincial. This classification captures spatial, but not temporal, dimensions of migration. Temporal issues could be captured through resident registration that includes (i) permanent registration (i.e. non-migrants who have *household registration books*, or *ho khau* in Vietnamese), (ii) temporal registration (i.e. migrants who stay in destination areas more than one month, are residing independently or with relatives, and who have *temporary household registration books*, or *tam tru* in Vietnamese), (iii) floating registration (i.e. migrants who stay in destination areas less than one month, are residing in guest houses or temporary dwellings, and who are without temporary household registration books), and (iv) non-registration (i.e. migrants who have not registered in destination areas) (9). However, the classification based on the resident registration system does not provide a clear definition of migration duration. In other words, it does not identify a certain duration of migration. Therefore, the census completed its migration definition by adding ‘five years prior to the census’ to the definition. Future research on migration should use definition and classification of migration in the census as key criteria for identifying migrants.

The classification in the census fails to distinguish between organized and spontaneous migration; however, this is reasonable for the 1989, 1999, and 2009 censuses. Indeed, in Vietnam, most migration before 1986 was organized and sponsored by the government. This kind of migration involved resettlement of persons into newly developing rural areas—the new economic zones—and migration became associated with job relocation (31). Meanwhile, migration flows in Vietnam after 1986 have been mainly spontaneous as a result of the restructuring of the economy and the development of individual entrepreneurship, foreign investment, and industrial zones. Organized migration has only occurred in some provinces, and it is driven by national construction projects such as hydroelectric power plants (1).

Our analysis demonstrates that interprovincial migration accounts for half of the mobile population. Through our examination of consecutive censuses, three notable trends were observed in interprovincial migration in Vietnam during the last 25 years: 1) relative and absolute migration flows are increasing; 2) the male-female ratio of migrants has displayed an inversion, with higher proportions of women observed in 2009; and 3) the age of interprovincial migrants is decreasing.

These trends, especially for rural to urban migration, are very similar to those in Thailand and in other Southeast Asian countries (3, 32, 33). The growth of interprovincial migrant populations has been fostered by economic development policies, lack of jobs in rural zones, lack of employees in urban areas, rapid urbanization, and the easing of policies that formerly restricted migration (15). These developments coincide with macroeconomic transitions that have led to notable economic growth and poverty reduction but with benefits inequitably distributed toward urban rather than rural areas (4). These trends have triggered contemporary internal migration flows and, in particular, interprovincial flows. Rural-to-urban flows put pressure on existing urban infrastructure and social services such as housing, health care, electricity, water, and sanitation (34, 35).

The ‘feminization of migration’ was clearly observed in our time series analysis. In fact, there was an evident gender pattern among migrants participating in labor markets. The literature attributes this trend to a declining agricultural sector and greater job opportunities for people in urban areas and industrial zones (36). Job opportunities for young female migrants have increased in the urban informal sector, including domestic housekeepers, restaurant employees, karaoke bar workers, street traders, and—in the formal sector—in textile, footwear, and garment factory jobs that favor women as a cheap, reliable, and ‘nimble-fingered’ workforce (37, 38). Meanwhile, men have taken more jobs in heavy industry such as iron and steel processing, mining, chemical processing, and electronic assembly (37, 39).

The interprovincial migrant population is also getting younger, especially among the female segment of the population. Migration populations from rural to urban areas have the highest concentration of young adult migrants. The flows of young migrants to urban areas also contribute largely to the economic development of those urban areas (35). This, in turn, exacerbates the divide between rural and urban zones at the provincial level. Within urban areas, average incomes of rural-to-urban migrants have been much higher than the national poverty standard; however, after migrants pay for room rent, electricity, and water, and they send financial support to their families in rural areas, their remaining available budget for food and other fundamental living items is small.

Young migrants are often healthy and better able to meet job requirements and working conditions (15). Some authors refer to this as the ‘healthy migrant effect,’ which implies that migrants seem to be healthier than non-migrants. However, Kristiansen et al. stated that the effect would fade out over time because migrants are exposed to many health risks (40). The World Health Organization (WHO) also found that migrants are more susceptible and vulnerable to ill-health effects and have more limited access to health care (i.e. they seek care more rarely or cannot pay for it) (41).

Most migrants in Vietnam have a secondary school education or less. Therefore, they usually work in manual labor jobs that are associated with low salaries, long working hours, no health insurance, and huge work pressures. Limited education can also block access to important social and health information, particularly about reproductive health. Indeed, Van Landingham (2003) indicated that rural-to-urban migrants cope with negative effects in almost all aspects of health such as physiology, psychology, sentiment, and so on (42). The 2004 Vietnam Migration Survey also identified several migrant-associated health problems including poor general health status, low use of health care services, and lack of knowledge about reproductive health and sexually transmitted infections (STIs). The majority of female migrants aged 20–29 (representing nearly 30% of total internal migrants), for example, displayed important misconceptions regarding reproductive health infections (RTIs), STIs, and HIV/AIDS (43). Lack of information poses several (health) risks to migrants and makes the human resource pool more vulnerable (43). More appropriate and adaptable health services for migrant populations clearly require more robust interpretations of the dynamics and characteristics of the relevant populations.

The results indicate that provincial characteristics of urbanization and monthly income per capita are associated with in-migration. Although this provides information on how socioeconomic factors relate to migration trends, it is important to acknowledge that the processes of migration, urbanization, and economic development are endogenous and go ‘hand-in-hand’ (1). In-migration flows contribute to increases in urban populations and to indicators of urbanization. In addition, rapid urbanization and structural reforms bring employment opportunities, which influence the acceleration of interprovincial, and especially rural-to-urban, migration. As a result, linear causal inference may be problematic and conceptually limited. In the literature, urbanization has been examined using quantitative and qualitative indicators (28); this analysis considers the proportion of the population living in urban areas (i.e. proportion of urban population) as the single indicator of urbanization due to limitations of secondary data. Hence, future research on urbanization and migration should consider other qualitative indicators such as improvements in living standards, changes in socioeconomic patterns, and diversity of cultural patterns.

Similarly, and with regard to the relationship between provincial income and in-migration, it may be inferred that in-migration contributes to the socioeconomic development of provinces; however, empirical evidence has shown that economic development is one of the strongest pull factors for in-migration (3, 4, 6, 13, 44, 45). That ‘pull’ effect was clearly demonstrated in some provinces in Vietnam such as Hanoi, Ho Chi Minh City, Binh Duong, Da Nang, and Dong Nai. In addition, the *Doi Moi* policies that encourage economic growth have enabled the development of large industrial zones that have led to increased urbanization (4). Binh Duong province is a typical example. Until 1997, it was a rural area belonging to Song Be province. After *Doi Moi*, Binh Duong attracted huge industrial capital, developed large industrial zones, and experienced rapid economic and urban growth and subsequent high monthly incomes. These developments made it an ideal destination for migrants. Indeed, this study found the highest in-migration rate to be in Binh Duong, compared with other urban areas. Some exceptions to this assertion exist, however. Lai Chau, for instance, is neither an urbanized nor high-income area, and yet its in-migration rate is quite high. This trend can be explained in terms of the government-organized migration of rural communities who lost their land after the construction of hydroelectric plants or other economic development projects. According to the 2005 report of the Asian Development Bank, about 78,000 people from Dien Bien, Lai Chau, and Son La were involved in a resettlement plan that allowed for the construction of the Son La hydroelectric power plant. In these ‘organized migration’ flows, most of the migrants are ethnic minorities. Of the relocated population, 60–90% were ethnic Thai (46). In the 2009 census, 6% of total migrants in Vietnam were minorities and most moved with organized migration or delocalization programs (28).

Kon Tum and Dak Nong are other notable exceptions. These new economic zones were organized by the government many years before *Doi Moi*. These two provinces are still ‘capitals’ of cash crops such as coffee. This has attracted migrants from other provinces, especially rural areas. In contrast to rural-to-urban migrants, most migrants moving to Kon Tum and Dak Nong were accompanied by their family and intend to stay there permanently.

It is important to note that the data in this study came from three sources: the 1989 national census capturing 5% of the population, the 1999 national census capturing 3% of the population, and the 2009 national census capturing 15% of the population. Although we strictly used the expansion factors for each census provided by the General Statistical Office, biases can exist in the data due to various weighting schemes used in the different nationally representative data sets. In addition, our estimates of interprovincial migration rates were based on movements 5 years prior to the census and thus exclude short-term, temporary migrants, circular migrants, and those who move without any registration. As a result, the actual number of interprovincial migrants may be higher than our estimates suggest. Those ‘uncounted’ populations may be particularly vulnerable (e.g. those who move without registration papers cannot access services at their destination). Furthermore, these migrants commonly work in informal sectors without employment contracts and insurance. Information about these ‘hidden or uncounted’ populations is not yet available. The goal of future research should be to provide more information about these populations in order to facilitate policies and interventions that take these groups into consideration.

## Conclusion

The analysis presented here is an attempt to describe and understand characteristics and trends of interprovincial migration and to discuss the relationship among migration, urbanization, and income per capita. Our analysis has also shown that interprovincial migration flows in Vietnam have changed dramatically over time and that these changes are characterized by an increase in relative and absolute migration flows, an inversion of the male-female ratio with higher proportions of women in 2009, and a decrease in the average age of migrants. These trends reflect unequally growing labor markets in Vietnamese provinces and imply improvements in infrastructure in these areas. Moreover, the trends challenge the national health system to ensure access to health care and health insurance for migrants and to design health services to cater to these populations.

There is a crude relationship between provincial socioeconomic status (monthly income per capita and proportion of urban population) and in-migration rate. Provinces with a high monthly income per capita and a high proportion of urban population are more likely to have a higher in-migration rate than other provinces. Further studies should examine these relationships in more depth. In addition, the development of industrial zones, the expansion of cities, and large national construction projects have a critical relationship with migration. Policymakers should consider these factors in developing economic programs to ensure that there is equitable access to public goods and that the needs of migrants are met (particularly younger females who account for a large segment of the migrant population).

## References

[1] UNDP (2010). Internal migration: opportunities and challenges for social-economic development in Vietnam. UN Publications by Agency.

[2] Deshingkar P (2006). Internal migration, poverty and development in Asia. Promoting growth, ending poverty Asia 2015.

[3] Guest P, Tienda M (2006). Bridging the gap: internal migration in Asia. Africa on the move: African migration and urbanisation in comparative respective.

[4] Phan D, Coxhead I (2010). Inter-provincial migration and inequality during Vietnam's transition. Journal of Development Economics.

[5] UNDP (2009). Overcoming barriers: human mobility and development. Human development report.

[6] Phuong T, Tam NTMT, Nguyet TN, Remco O (2008). Determinants and impacts of migration in Viet Nam.

[7] Skeldon R (1997). Rural-to-urban migration and its implications for poverty alleviation. Asia-Pacific Population Journal.

[8] GSO (2006). The 2004 migration survey: internal migration and related life course events.

[9] Vietnam National Assembly (2006).

[10] Le DB, Nguyen LT (2011). From countryside to cities: socioeconomic impacts of migration in Viet Nam.

[11] Brauw AD (2007). Seasonal migration and agriculture in Viet Nam. The Food and Agriculture Organization of the United Nations: Paper prepared for presentation at the FAO-sponsored workshop on ‘Migration, transfers and household economic decision making,’ Rome.

[12] UNAIDS (2001). Population mobility and AIDS. UNAIDS technical update.

[13] Djamba Y, Goldstein S, Goldstein A (1999). Permanent and temporary migration in Viet Nam during a period of economic change. Asia–Pacific Population Journal.

[14] GSO (2009). The 2009 Viet Nam population and housing census of 00.00 hours 1st April 2009: expanded sample results.

[15] Liem N (2009). Youth internal migration and development in contemporary Vietnam. Workshop on migration, development and poverty reduction.

[16] FHI (2001). HIV/AIDS behavioural surveillance survey Viet Nam 2000.

[17] Lam H, Dan N, Lai P (2005). Some risk behaviours to HIV/STDs of seafarers, at transportation and fishing sites. Thai Binh, Journal of Practical Medicine.

[18] Nghi N (2010). Status of workers at industrial zones in Tien Giang. Journal of Numbers and Events.

[19] Anh LTK, Lien PTL, Hung NT (2011). Inter-provincial migrants working in industrial areas: living conditions, activities, and the use of health services. Journal of Practical Medicine.

[20] Anh LTK (2012). Situation of living conditions and health service utilization of migrants in Sai Dong industrial zone, Long Bien, HaNoi 2011. Journal of Military Phamaco–Medicine.

[21] GSO (1991). Vietnam population census—1989: completed census results.

[22] GSO (2001). The 1999 Vietnam population and housing census—major findings.

[23] Central Population and Housing Census Steering Committee (2010). The 2009 Vietnam population and housing census–major findings.

[24] GSO (2009). The 2009 Vietnam population and housing census of 00.00 hours 1st April 2009: implementation and preliminary results. The 2009 Vietnam population and housing census.

[25] GSO (2008). Results of the survey on household living standards 2008.

[26] Vietnam goverment (2011). National administrative map.

[27] Vietnam government (2009). National administrative map.

[28] GSO (2011). Vietnam population and housing census 2009. Migration and urbanization in Vietnam: patterns, trends, and differentials.

[29] Vietnam goverment (2001). Decree No. 72/2001/ND-CP 05 October 2001 of Goverment of Socialist Republic of Vietnam on classification of city.

[30] GSO (2010). The 2009 Vietnam population and housing census: some key indicators. The 2009 Vietnam population and housing census.

[31] Djamba G, Goldstein S, Goldstein A (1999). Permanent and temporary migration in Vietnam during a period of economic change. Asia–Pacific Population Journal.

[32] Hugo G (1993). Migrant women in developing countries. Internal migration of women in developing countries.

[33] Perjaranonda C, Santipaporn S, Guest P (1995). Rural-urban migration in Thailand. Trends, patterns, and implications of rural-urban migration in India, Nepal, and Thailand. Asian Population Studies Series.

[34] Thanh L (2006). Migration and the socio-economic development of Ho Chi Minh City (Viet Nam).

[35] Liem N, White M (2007). Health status of temporal migrants in urban areas in Viet Nam. International Migration.

[36] Anh D, Tavoli C, Thanh H (2003). Migration in Vietnam: a review of information on current trends and patterns, and their policy implications. The regional conference on migration, development, and pro-poor policy choices in Asia.

[37] Lim L (1993). The structural determinants of female migration. Internal migration of women in developing countries.

[38] Resurreccion B, Ha T (2007). Able to come and go: reproducing gender in female rural–urban migration in the Red River Delta. Population, Spaceand Place.

[39] Anh DN (2006). Internal migration in Viet Nam: solutions for rural development. Sociological issues in renovation period.

[40] Kristiansen M, Mygind A, Krasnik A (2007). Health effects of migration. Dan Med Bull.

[41] WHO (2010). Health of migrants—The way forward. Report of a global consultation.

[42] Van Landingham M (2003). Impacts of rural to urban migration on the health of working-age adult migrants in Ho Chi Minh City, Vietnam. Conference on African migration in comparative perspective, Johannesburg, South Africa.

[43] GSO (2006). The 2004 migration survey: migration and health.

[44] Kundu A (2009). Urbanization and migration: an analysis of trends, patterns, and policies in Asia. Human Development Research Paper 16.

[45] Anh ND, Goldstein S, McNally J (1997). Internal migration and development in Vietnam. International Migration Review.

[46] ADB (2005). Implementation of the environmental management plan for the Son La hydropower project. Technical Assistance Report, Project Number 39537.

